# Development and Validation of a Recipe Method for Doughs

**DOI:** 10.3390/foods7100163

**Published:** 2018-10-02

**Authors:** Adriana Lezama-Solano, Edgar Chambers

**Affiliations:** Center for Sensory Analysis and Consumer Behavior, Kansas State University, Manhattan, KS 66502, USA; alezama@ksu.edu

**Keywords:** doughs, recipes, Think Aloud, consumers, qualitative research

## Abstract

Recipes have a great impact on consumers’ behavior in the kitchen; building a recipe requires the understanding of the potential user. The objective of this study was to develop and evaluate different recipe styles for the preparation of doughs by understanding people’s descriptions of these. Two qualitative studies were performed (43 wheat and 50 corn dough preparers). During interviews, participants described the preparation process of the doughs using the Think Aloud technique. Finished doughs were described as not sticky, soft, and pliable. Based on these descriptions, five recipes were created: not detailed, detailed, very detailed, paragraph-form, and ‘with images’. Recipes were validated in two online surveys (total *n* = 600), where respondents evaluated the easiness, likeability, likelihood of using, helpfulness, and amount of information. Respondents considered the recipe with images as easier and more helpful. The very detailed recipe was considered more difficult, less helpful, and was liked less than the other recipes. Understanding and identifying the terms and techniques people use is a good way to communicate how to prepare a food product and can be used to develop and improve recipes. However, the format in which the recipe is presented is an important factor considered by users when following recipes.

## 1. Introduction

Currently, families in the United States are more likely to eat at home, which has increased the use of recipes as a guide for cooking preparations. A survey from 2012 shows that 67% of home cooks had used a recipe at least once in the last month [1]. This is especially influenced by Millennials, who are using recipes for cooking at least once a week [2].

Recipes can be defined as sets of directions that tell the user how to cook and prepare a food product. They are usually derived from traditions where cooking was learned by imitation. Recipes frequently have an ingredients list followed by cooking directions [3]. However, there are some other key factors that users consider important in a recipe, such as cooking temperatures, possible variables during the process, and expected outcomes (including sensory characteristics of the final product) [3,4,5,6].

Recipes have a great impact on consumers’ behavior in the kitchen. Various authors [7,8] determined that users have better food safety behaviors when recipes have food safety instructions compared to occasions when recipes do not have this type of instructions. Therefore, providing accurate information on how to prepare a product is crucial for consumers’ understanding, satisfaction, and safety [6].

Building a recipe requires food science knowledge, as well as an understanding of the potential user. The writer needs to be able to communicate to the reader or preparer the best way to make the food product. Directions should be easy to read and follow, should be written in layman’s terms, and should not be cluttered [9,10]. Bielunski [11] directed a survey to explore what consumers want in recipes. Respondents mentioned they liked recipes that seem easy to prepare; the easiness of the recipe was evaluated based on the number of ingredients, preparation time, and overall readability. 

There are multiple formats in which a recipe can be written. Using focus groups, Levis et al. [6] studied three different recipe styles: paragraph-form, numbered step-by-step, and a graphical/text format. Results showed that consumers preferred the step-by-step format since it was easy to read and follow. The graphical/text format was also liked because it was eye-catching, easy to follow, and gave them confidence, but consumers commented that a new cook would not be able to follow just graphical information because it provided insufficient information. Bielunski [11] studied people’s impressions of paragraph-form recipes (single and multiple paragraphs) and numbered and bulleted recipes. Participants mentioned they liked recipes where the ingredients were broken down and where preparation steps were numbered or bulleted. Interviewees also preferred recipes with short and concise directions that were still specific [11,12].

Anecdotal evidence and the authors’ observations (including working in recipe development at a flour manufacturer) suggests that dough making is not considered to be “easy” by consumers. Terms such as “knead until ready” are vague and provide no obvious clues as to what “ready” is. 

Thus, the objective of this study was to develop and evaluate different recipe styles (not detailed, detailed, very detailed, paragraph-form, and with images) for the preparation of doughs by understanding how people at home make and describe these products.

## 2. Materials and Methods

This study was approved by the Institutional Review Board (Human Subjects) of Kansas State University.

### 2.1. Qualitative Study

#### 2.1.1. Subjects’ Recruiting 

Two observational studies to determine how consumers prepare and describe wheat and corn doughs were conducted through personal interviews in two locations: Manhattan, KS, USA, (43 wheat dough preparers) and Guadalupe, San José, Costa Rica (50 corn dough preparers).

To be part of the studies, preparers had to be over 18 years old and not professional bakers or chefs. For the wheat dough study, participants were bakers of yeast breads or pizza from scratch. For the corn dough study, participants were cooks of tortillas, empanadas, or bizcochos (a type of crunchy corn-based ring made from corn dough) from scratch. Participants were told that the study required them to agree to prepare a dough from scratch while they described their experience to a researcher. 

For the wheat dough study, participants were recruited via RedJade Sensory Software using the consumer database of the Center for Sensory Analysis and Consumer Behavior at Kansas State University. Therefore, most of the participants were from the Manhattan, KS area. The corn dough study was conducted in Costa Rica, where participants were recruited through an external marketing agency; screening was done in person with consumers in their database. Most of the participants were from regions close to Guadalupe, Costa Rica. The recruiting and interviews in Costa Rica were done in Spanish and the information collected was later translated into English.

#### 2.1.2. Participants’ Demographics

Table 1 shows the demographic information of preparers who participated in the studies. 

#### 2.1.3. Dough Preparation Sessions

Home-style kitchens were used in each of the locations. Participants in Manhattan were asked to prepare a wheat dough for a yeast bread, and in Costa Rica, a corn dough for tortillas. A variety of equipment was supplied. This included bowls, spoons, spatulas, and measuring spoons and cups; mixers were not provided. Wheat dough preparers were given containers of commercial all-purpose wheat flour, salt, instant yeast, vegetable oil, and water. Corn dough preparers were given yellow corn flour, salt, and water. Each of the ingredients was measured both prior to use and post-use to determine the actual amount of ingredients used by the consumers. Table 2 shows the amount of each of the ingredients given to consumers. 

A basic, not detailed, recipe was given to participants as guidance; however, they were told they did not need to follow it since they were encouraged to prepare the dough as they usually would do at home.

In order to obtain descriptions of the doughs and the technique used by participants, the Think Aloud technique [13] was used. This technique required participants to speak out their thoughts while preparing the dough. This technique produces direct information of the ongoing thinking process while the task is being performed; rather than asking questions about a past process [14,15,16,17]. Training in the technique, as described in prior research [18], was done to get the consumers comfortable using the technique, and the training included a size arrangement exercise and a figure-color matching exercise. Then, prior to dough preparation, participants were instructed to think aloud while preparing the dough. Each participant verbally described his or her strategy and process for deciding how to make the dough. 

The dough preparation sessions were video recorded. Moderators took pictures of the ready to use corn and wheat doughs. 

### 2.2. Post Session Interview

A brief interview was done with participants after the completion of the dough preparation. Participants were asked how similar the dough preparation was during the sessions to the way they would do it in their own kitchens. Then, they were asked to describe, just using words, how they decided when the mixing stage (in both studies) and the kneading stage (only in the wheat dough study) were done and the dough was ready for use (i.e., ready dough). 

### 2.3. Moderators

Moderators participated in a training session and a practice session; seven assessors moderated the wheat dough preparation sessions. In the corn dough preparation, two moderators guided the sessions. A detailed protocol was provided to each of the moderators, as well as an observational worksheet.

### 2.4. Recipes Validation

Based on descriptions given by consumers during the dough preparation sessions, five different styles of recipes for each type of dough were developed. The styles studied were:

1. Not detailed recipe: same as the one given to experienced consumers during the dough preparation sessions; written in a step-by-step format.
2. Detailed recipe: recipe that included details, but not as many as the very detailed recipe. This recipe was written in a step-by-step format.
3. Very detailed recipe: recipe that included a complete and exhaustive description of the preparation of the dough and the ready dough; written in a step-by-step format.
4. Paragraph-form; based on the detailed recipe, the process was described in paragraphs.
5. Images; also based on the detailed recipe. The process was written using pictures taken from the dough preparation sessions and some captions to describe each of the steps.

All the recipes included the yield of the recipe, an initial setup that instructed readers to wash their hands, and baking/cooking steps. These steps were presented in the same way for all the recipes evaluated. The recipes can be found in Appendix A. 

#### 2.4.1. Online Survey

An online survey to collect consumers’ impressions of each of the recipes was conducted using Qualtrics software (Qualtrics, Provo, UT, USA) licensed for Kansas State University. Participants from around the U.S. were surveyed; 300 for wheat dough and a different 300 for corn dough. Each questionnaire included three sections. The first was a screener that respondents had to pass in order to participate in the survey. Second were the recipe questions (recipe validation), which recorded respondents’ impressions of each of the recipes. For each recipe, respondents were asked to rate how easy they thought the recipe was, how likely they were to use the recipes at home, how much they liked the instructions, and how helpful the format of the recipe was. They also answered Check-all-that-Apply (CATA) questions regarding what they liked and what they did not like about each recipe. The last section was a demographics questionnaire, where consumers were asked their gender, age range, and ethnicity. 

#### 2.4.2. Respondents

To participate in the survey, respondents needed to be over 18 years old and not professional bakers or cooks. They had to rate their own cooking abilities as novice, basic, or average. They also had to answer how often they had prepared corn tortillas or yeast breads (not using a bread machine) from scratch. To qualify, consumers could not have made yeast bread or corn tortillas more than twice in the past five years. However, participants needed to be interested in learning how to make corn tortillas or yeast breads. Table 3 shows the demographics of participants in the study. 

### 2.5. Data Analysis

#### 2.5.1. Qualitative Study: Dough Preparation Sessions

A transcript of each of the sessions was built using the video recordings and the written notes on the observational worksheets. Transcripts were edited to get the information needed to build the recipes: the amount of ingredients (g), measurement technique (household/volume, weight, a combination of both, or neither), and water temperature (°C). For the mixing and kneading, the following information was collected: time, utensils used, the technique applied, and attributes used to describe the dough. For the attributes and the techniques, common or similar terms were grouped together into a category. 

#### 2.5.2. Recipe Validation: Online Survey

In each study, the data on the easiness of the recipes, how likely participants were to use the recipes at home, how they rated the amount of information, how much they liked the instructions, and how helpful they found the format of the recipes was analyzed using analysis of variance (ANOVA). Significant differences (*p* ≤ 0.05) across recipes were evaluated using Tukey’s HSD test. Pearson’s correlation coefficient was used as an exploratory tool to determine significant associations among responses. Respondents were clustered using K-means based on the liking scores of the instructions to analyze the differences/similarities among groups. 

CATA questions were analyzed using Cochran’s Q tests. To illustrate the relationship between recipes and the parameters tested, a correspondence analysis (CA) was performed considering chi-square distances; based on this, a symmetric plot was built. 

All the analyses were done using XLSTAT-Sensory, sensory analysis statistical tools in Excel (Version 19.4 2017.06.19, Addinsoft, New York, NY, USA).

## 3. Results and Discussion

### 3.1. Preparation Process

#### 3.1.1. Measurement of Ingredients

Measurement techniques used by consumers when preparing the doughs are shown in Table 4. During the wheat study, 86% of preparers used the household/volume method, while only 18% of corn preparers used this technique. Weight was the technique used the least in both studies, with 5% in the wheat study and 2% in the corn study. For the corn tortillas, most consumers did not measure ingredients; they estimated and dumped using eyes and hands to decide how much of each ingredient to use. Recipes were built using household/volume measures since it was the most common technique used by preparers. This technique is not as accurate as weighing ingredients, especially for solid food products like flour. However, it is commonly preferred during cooking in many countries since it is faster, and accuracy might not be as important while cooking [19].

Not measuring ingredients was usual among participants; 84% of wheat and 88% of the corn dough preparers did not measure at least one of the ingredients. Flour was the main ingredient not measured: 74% of wheat dough preparers and 80% of corn dough preparers did not measure the amount added. During the corn dough preparation, most of the preparers did not measure any of the ingredients. Not measuring ingredients is related to the cooking abilities of the preparers and their knowledge of the ingredients. Participants were not asked to rate their own cooking skills; however, 84% of corn dough preparers mentioned that they prepare corn dough products daily or weekly. Previous studies show that a high frequency of food preparation is an indication of high cooking abilities [20,21].

Water was not measured by 88% of corn dough preparers in contrast to 9% of wheat dough preparers. The salt was not measured by 82% of the corn dough preparers, but all the wheat dough preparers measured it. 

Table 5 shows the amount of each ingredient used by dough preparers. During the wheat dough study, consumers used an average of 326 g of flour. Between 191 g and 378 g of water was used, with an average of 251 g. The average for both salt and yeast was 7 g. The amount of oil was between 0 g and 112 g. For the corn study, preparers used between 43 g and 500 g of corn flour, with an average of 158 g; the amount of water was an average of 229 g. The salt ranged between 0 g and 52 g, with an average of 6 g. Since most of the corn participants did not measure the ingredients, they were asked for the yield from the dough prepared, which on average, was three tortillas. 

Recipes were built based on the average amount of each ingredient used by dough preparers. In both studies, the amount of water used by preparers was more than the amount used in other studies. For the wheat dough, the AACC [22] method suggests 47% (bakery percentage) of water, while Curic et al. [23] suggest 58%. Contreras-Jimenez et al. [24] reported a water absorption value for corn flour between 80% and 111%. However, the amount added varied depending on the type of flour and the user preferences, among other things. 

Regarding the temperature of the water, 49% of wheat dough preparers used warm water between 30 °C and 66 °C, with an average of 44 °C. In the corn dough preparation, 22% of preparers used water between 32 °C and 71 °C, with an average of 46 °C. Even when not all the preparers used warm water, the literature suggests that the temperature of the dough is a key factor to ensure uniform processing conditions and the final product quality. For bread making, Cauvain and Young [25] recommend a temperature of 30 °C; commercial brands’ packages recommend a temperature between 49 °C and 55 °C for instant yeast. During yeast bread preparation, warm water guarantees better conditions for the yeast development [25]. For the preparation of tortillas, less information is available; however, warm temperature improves dough performance in the next stages (like sheeting or forming). Due to the importance of the temperature during dough preparation, recipes were written using warm water, even when most of the preparers in the qualitative study used room temperature water.

#### 3.1.2. Measurement of Ingredients

When preparing the doughs, 33 of the 43 wheat preparers and 15 of 50 the corn dough preparers mixed the dry ingredients before adding the water. During the wheat dough study, preparers took between 6 s and 2.75 min, with an average of 33 s. The corn dough preparers took between 5 s and 1.25 min, with an average of 21 s. Mixing wet and dry ingredients took between 53 s and 9.15 min for wheat dough preparers, with an average of 3.78 min. Corn dough preparers took between 44 s and 5.75 min, with an average of 2.7 min. The kneading stage, which only took place during the wheat dough preparation, took between 57 s and 10.53 min, with an average time of 4.55 min. Average times were included in the recipes for the main stages. Previous studies show that food preparers like the addition of times in recipes [3,11].

Table 6 shows the utensils used by dough preparers. During the wheat dough mixing stage, spoons (wooden and metal) were the most commonly used; 63% of preparers used them while mixing the dry ingredients and 72% while mixing wet and dry ingredients. Preparers also used their hands to mix ingredients, 5% used them when mixing dry ingredients, 7% when mixing dry and wet ingredients, and all of them during kneading. The use of hands as a utensil was more common in the corn study, where 96% of preparers used them to mix the wet and dry ingredients. Metal spoons were also used by 14% of people while mixing dry ingredients, and 16% while mixing wet and dry ingredients. Spoons represent one of the most common utensils used by people in their kitchens, as reported by Wang and Worsley [26]. The use of the hands might be related to the easiness to prepare the products by direct contact with them. Additionally, as the results will show, the main criterion to decide if the dough is ready is through the texture perceived with the hands. 

The techniques that preparers used to mix the ingredients and knead the dough are shown in Table 7. Circular motions and from the edges to the center were the most common techniques used when mixing the dry ingredients in both studies. During the mixing of all the ingredients, for both doughs, mixing all the ingredients together, circles/stirring, scraping the bowl, and adding water a little bit at a time were the most common techniques used. Pressing, pushing, or squeezing the dough was also a common technique when preparing the corn dough, as well as during the kneading of the wheat dough. Other techniques commonly mentioned by preparers during kneading were folding the dough and stirring. 

The main goal of the mixing and kneading stages is to input energy into the mix. This energy input helps the gluten development, incorporation of air, and formation of an extensible dough. In both doughs, the energy contribution helps to obtain a dough from the mixture of all the ingredients [27]. Pressing, pushing, stirring, and folding are common techniques for these stages, according to the literature [28].

#### 3.1.3. Description of a “Ready Dough”

Attributes given by preparers to describe the ready dough are presented in Table 8. One ball, mixed in, and homogeneous were common attributes used to describe the wheat doughs after mixing. This was expected since the main objective of mixing is to incorporate all the ingredients together, i.e., to homogenize. [25,27]. Participants also mentioned some similar attributes to describe the mixture of the dry ingredients; the main attribute mentioned was all mixed in, which included the yeast being evenly spread.

Sticky was one of the main attributes mentioned after mixing the wheat dough, elastic was also common in this dough after kneading, and not sticky was mentioned in both studies to describe the ready doughs. Rheological studies show that before kneading, dough is more sticky and wet; these characteristics decrease during kneading and other characteristics like cohesiveness and elasticity arise [29,30]. The adhesiveness of the dough is another textural parameter often measured in rheological studies; this relates to what preparers called stickiness [31].

Participants did not mention cohesiveness, a common term used in the instrumental texture analysis of doughs. Lawless and Heymann [32] indicate that cohesiveness is a complex and very technical attribute which might be too specific for regular consumers with no further training or knowledge [30,33].

Most of the attributes mentioned by preparers in both doughs preparations relate to the texture of the dough, specifically to mechanical textural characteristics. These characteristics represent how the dough reacts to stress like pushing, pressing, or stirring [34]. The results show how doughs are an example of a food product where texture is more important than flavor [35]. However, 70% of corn dough preparers still considered the saltiness as a key component to decide if the dough is ready or not, contrary to wheat dough preparers that did not taste the dough. 

The descriptions obtained by this study can be compared to the study done in 1937 by David Katz. Body, a common attribute used to describe doughs in his study, was not a term used by participants in the current studies. However, Katz related other attributes to the body of doughs, such as stickiness and elasticity, while dough preparers in the present study did mention these attributes [36,37,38].

A common term mentioned by consumers was not too soft but not too hard. Szczesniak [38] points out this as one of the main limitations when studying texture, since there are no clear and stated boundaries between these attributes, firm and hard. 

### 3.2. Recipe Validation

**Figure 1:**
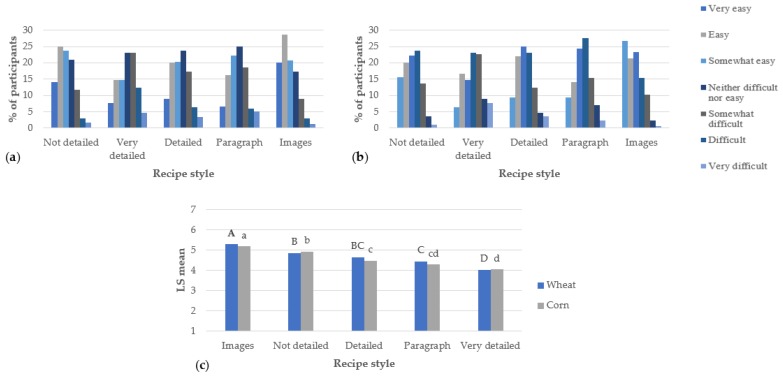
Easiness of the recipes presented to respondents (per study, *n* = 300), including (**a**) wheat study, (**b**) corn study, and (**c**) ANOVA. For the ANOVA, statistical comparisons are within wheat and corn recipes: bold, uppercase letters represent the wheat recipes; not bold, lowercase letters represent the corn recipes. Different letters among studies represent significantly different means (*p* < 0.05).

The results of the Qualtrics surveys for the recipes are shown below. Figure 1 shows consumers’ perceptions of the ease of the recipes. In both studies, most of the people considered the recipes with images easy, very easy, or somewhat easy. The ANOVA showed that the scores were significantly higher for these recipes in both surveys. Levis et al. [6] found similar results in their study, where participants considered recipes with images easy to read and follow. In other studies, the step-by-step format was also considered easy by evaluators [11,12]. The step-by-step format was the one used for the very detailed, detailed, and not detailed recipes in this research. However, the very detailed recipe was considered the most difficult in both studies. The not detailed recipe was the longest of the recipes in both surveys, and this finding suggests that the format does not affect the perceived easiness of the recipe as much as the length of it. Previous studies suggest that the overall readability and the length of the recipes influence how recipe users perceived the ease of recipes [11]. 

In the corn study, there were no significant differences between the very detailed and the paragraph format recipes. Levis et al.’s [6] research found that participants did not consider the paragraph-form recipe easy since it required them to reread more often than an image or step-by-step format. 

**Figure 2:**
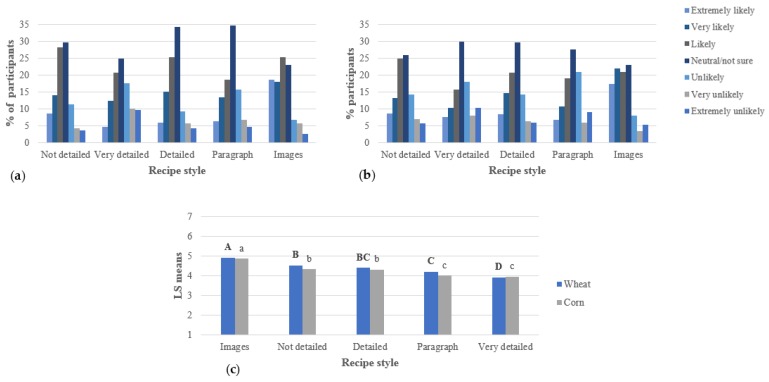
Likelihood to use the recipes presented to respondents (per study, *n* = 300), including (**a**) wheat study, (**b**) corn study, and (**c**) ANOVA. For the ANOVA, statistical comparisons are within wheat and corn recipes: bold, uppercase letters represent the wheat recipes; not bold, lowercase letters represent the corn recipes. Different letters among studies represent significantly different means (*p* < 0.05).

Similar results were shown when consumers were asked how likely they were to use the recipes at home, and Figure 2. shows these results. Respondents were significantly more likely to use the recipes with images, while the paragraph format and the very detailed recipes were the least likely to be used. Bielunski [11] found that the perceived easiness of the recipe is a key factor that determines people’s likelihood to use the recipes at home. In both studies, the not detailed recipe, which is the shortest one of the step-by-step recipes, was the second most likely to be used by respondents.

**Figure 3:**
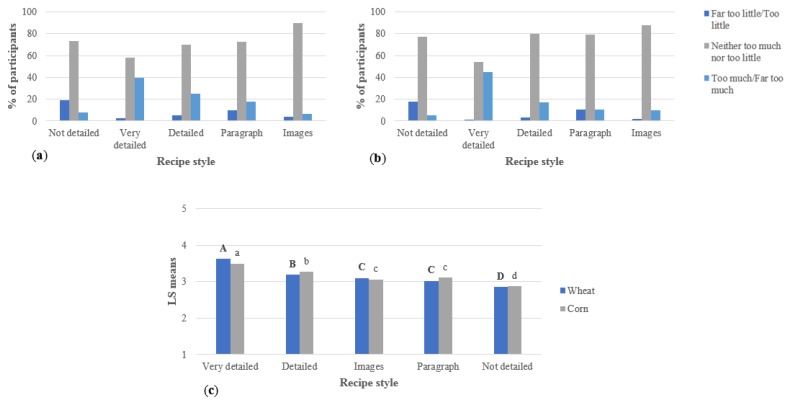
Perceived amount of information in the recipes presented to respondents (per study, *n* = 300), including (**a**) wheat study, (**b**) corn study, and (**c**) ANOVA. For the ANOVA, statistical comparisons are within wheat and corn recipes: bold, uppercase letters represent the wheat recipes; not bold, lowercase letters represent the corn recipes. Different letters among studies represent significantly different means (*p* < 0.05).

Figure 3 shows consumers’ impressions of the amount of information in the recipes. The ANOVA shows that for both surveys, the respondents considered the very detailed recipe to have significantly more information than all the other recipes, while the not detailed recipe had significantly less. The detailed recipe and the image and paragraph format recipes were all written with the same base, but the paragraph format allows presentation of the information in a more compact or cluttered way, while the use of images allows the use of fewer words and presents some additional, not written, information through the images [6]. This explains why the detailed recipe was considered to have more information than the other two recipes mentioned. 

For the not detailed recipe with a lack of details and descriptions, the average results show that respondents considered this recipe to have “far too little/too little” information. As mentioned, shorter recipes usually are perceived as easier for participants. However, the recipe with images, considered the easiest one for participants, was not rated as “far too little/too little” as much as the not detailed recipe [3,11].

For all the recipes in both surveys, more than 50% of participants considered the amount of information as “neither too much nor too little”. The percentage was even higher in the recipe with images (close to a 90% on both surveys) and presented the lowest value in the very detailed recipe (54% in the wheat study and 58% in the corn study). These results make the average values very close to each other, ranging from 2.8% to 3.6%. However, a group of respondents considered the amount of information in some recipes “too little”, especially in the not detailed recipe, or “too much”, like in the very detailed recipe. These observations influence the averages reported and allow statistical differences to be obtained among recipes in both studies. 

**Figure 4:**
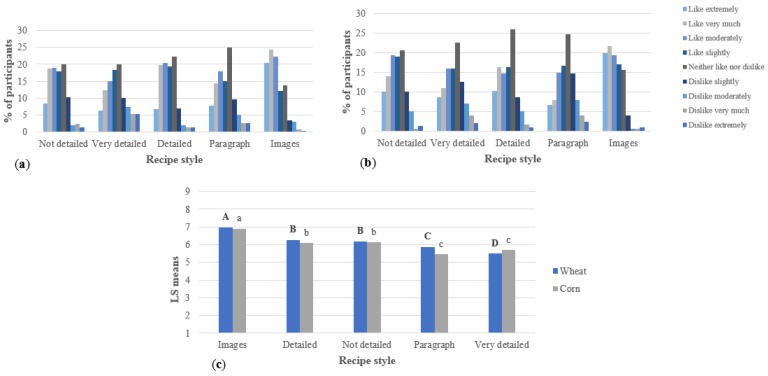
Likeability of the recipes presented to respondents (per study, *n* = 300), including (**a**) wheat study, (**b**) corn study, and (**c**) ANOVA. For the ANOVA, statistical comparisons are within wheat and corn recipes: bold, uppercase letters represent the wheat recipes; not bold, lowercase letters represent the corn recipes. Different letters among studies represent significantly different means (*p* < 0.05).

Respondents’ likeability towards the instructions is shown in Figure 4. The recipe with images was the one liked the most by respondents, and it was the only recipe where most of the respondents mentioned that they liked it very much. In both studies, the very detailed recipe was the one liked the least; however, in the corn study, it did not show statistical differences to the paragraph-form recipe. In Levis et al.’s [6] study, participants did not like the paragraph format because it required rereading more often compared to a step-by-step recipe or a recipe with images. They had to read the entire paragraph before cooking, and it was easy to miss some parts of the recipe. 

In previous surveys and studies, recipe users indicated that they liked specific recipes that tell them what to do with a vocabulary easy to understand by the naïve cooks [11,39]. The present study shows that, even when consumers want specific details and further explanations of some techniques, they do not like and do not want to use long, very detailed recipes since they are considered difficult.

**Figure 5:**
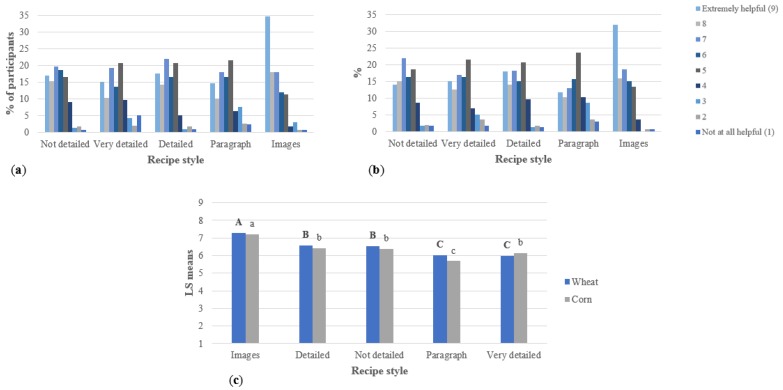
Helpfulness of the format of the recipes (per study, *n* = 300), including (**a**) wheat study, (**b**) corn study, and (**c**) ANOVA. For the ANOVA, statistical comparisons are within wheat and corn recipes: bold, uppercase letters represent the wheat recipes; not bold, lowercase letters represent the corn recipes. Different letters among studies represent significantly different means (*p* < 0.05).

Consumers found the recipe with images as the most helpful, as shown in Figure 5. As mentioned, this recipe was considered the easiest one, and it was the recipe that respondents liked the most and were more likely to use at home. Previous studies suggest that participants like this kind of recipe since they can pause on keywords and use the images as a guide that helps them picture the product [6,11,40]. The paragraph format was the one considered least helpful. The paragraph format recipe did not present a statistical difference compared to the very detailed recipe in the wheat survey.

The not detailed, the detailed, and the very detailed recipes were all written in the same format, step-by-step. Previous studies suggest this is one of the preferred formats by recipe users since it is easy to read and follow and participants could stop at keywords easier [6,11]. In the wheat survey, there was a statistical difference between very detailed recipes and the other two step-by-step recipes. This might be an indication that consumers’ evaluation of the helpfulness of the recipe is related to how much they liked the recipe rather than the actual format of it.

The results of the correlation test (see Appendix B) confirm that the how answers are mostly based on how much respondents liked the instructions of the recipes. For both studies, the Pearson’s correlation test showed high correlations (*p* > 0.6) between the likeability of the instructions and perceived helpfulness of the format, likeability of the instructions and ease of the recipes, likeability of the instructions and likelihood to use the recipes, and ease of the recipes and the likelihood to use them. Additionally, for the wheat survey, the format helpfulness and the likelihood to use the recipes were highly correlated. No additional correlations were found in the corn study. The amount of information was the only parameter that did not present a correlation with at least one of the other items evaluated.

**Figure 6:**
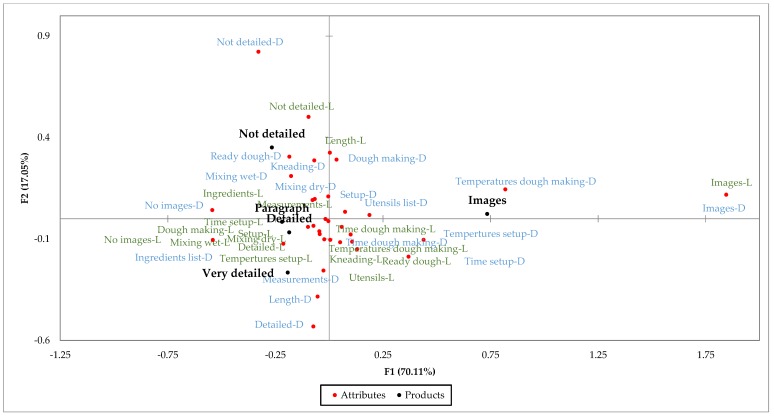
Correspondence analysis factor map representing five wheat recipes and 18 liking (L) and disliking (D) factors. This factor map represents 87.17% of the total variance, with factor 1 contributing to 70.11% and factor 2 covering 17.05% of the variance.

**Figure 7:**
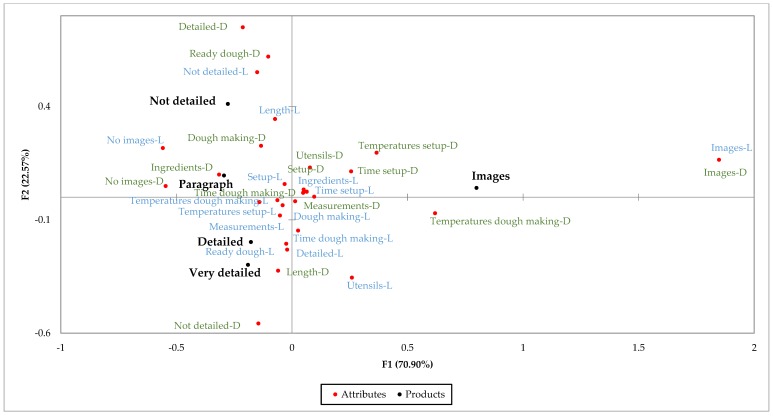
Correspondence analysis factor map representing five corn recipes and 15 liking (L) and disliking (D) factors. This factor map represents 93.47% of the total variance, with factor 1 contributing to 70.90% and factor 2 covering 22.57% of the variance.

Figure 6 and Figure 7 show what participants liked and disliked about each recipe based on the responses to the CATA questions. According to these plots, participants found some differences among recipes in terms of what they liked and what they did not like. 

As seen on the plots for both surveys, participants liked the presence of images on the recipes that had them, that the detailed and very detailed recipes were very detailed, and that the not detailed recipe was not very detailed. Nevertheless, these were disliking factors for other respondents. 

Based on the plots, the *X*-axis shows two groups of respondents: people that like the presence of images and people that like the absence of images. The number of people that liked the presence of images was close to 75%, and less than 10% mentioned they liked the absence of them. The plots for the disliking factors show similar results; about 5% mentioned that they disliked the presence of images, while about 50% mentioned that they disliked the absence of them. Based on the data collected, even when some people disliked the presence of images, these represent a very small group. These results confirm that the presence of images is an important factor in the evaluation of these recipes.

The *Y*-axis shows that some respondents liked the lack of details in the not detailed recipe, while others liked that the detailed and very detailed recipes were very detailed. Also, a group disliked the presence of details on the more detailed recipes, while another group disliked the absence of these in the less detailed recipes. Previous research mentions that recipe users like specific and detailed recipes. However, these results show there is a group of people that do not like the presence of details [12]. 

As mentioned before, the not detailed recipe is the same recipe given to dough preparers. During the dough preparation study, some of the participants, mostly wheat preparers, mentioned that the recipe was not clear and not a good guide for the process. However, participants in the survey mentioned they liked it because it was not very detailed and because of the length of it. Additionally, it was considered as one of the easiest recipes and more likely to be used at home (after the recipes with images). This suggests that results might be different when users prepare food products using the recipes compared to what they answered on surveys just by reading the recipes.

#### Cluster Analysis

For both studies, the K-means procedures found two clusters. For the wheat study, cluster 1 had 169 observations, and cluster 2 had 131 observations. The ANOVA showed that both clusters liked recipes with images the most. Both clusters liked the very detailed recipe the least, but in the second cluster, it did not present significant differences with the paragraph format and detailed recipes. Cluster 2 was also characterized by higher scores compared to cluster 1. 

In the corn study, cluster 1 had 161 observations, while cluster 2 had 139 observations. In both clusters, the recipe with images had significantly higher likability scores, while the paragraph format recipe was the one with the lowest likeability scores; however, in cluster 1 it did not present significant differences with the very detailed recipe. Like in the wheat study, the second cluster presented higher scores than cluster 1. The results of each cluster are presented in Appendix C.

### 3.3. Practical Applications

This research retrieves consumers’ terms and descriptions of doughs. Information collected helps to better understand a food product that has not been studied enough in the sensory field. The research includes descriptions of how regular home users manipulate doughs, important consumers’ attributes on corn and wheat doughs that can be applied to other type of doughs, and consumers’ overall experience when preparing doughs for yeast breads and tortillas. The results can be later applied for the future development of trained sensory panels of doughs, quantitative research for consumers, or explanations of the preparation process to dough makers in the industry and at home [41,42].

Participants in the surveys preferred the presence of images on the recipes presented. This information can be used in cooking or handling instructions on food packages to encourage and guide users on the preparation and use of the product. It also provides a guide on what is the best way to communicate with recipe users or food preparers. 

### 3.4. Limitations

The corn dough preparations are not as common in the United States (except for Latin American communities), which is why the corn dough study was performed in Costa Rica. However, the recipes were evaluated in the United States after the translation of the terms and descriptions. Cultural differences might exist between consumers’ description and perception of sensory terms, especially due to the translation process [43]. In the same way, preparers in the United States described the preparation and the ready dough, so some of these descriptions might not be applied in the same way in other parts of the world [44].

Furthermore, recipes were validated through a survey and not through a cooking exercise. Dough preparers had a different impression of the not detailed recipe compared to survey respondents. The results presented in the recipe validation stage could differ if the recipes were used in a cooking exercise; however, consumers typically look at a recipe before deciding whether to use it. It is unlikely that consumers would select a recipe that they thought was too complicated or that they did not think would be helpful.

## 4. Conclusions

Personal interviews where regular cooks prepare and describe the preparation of a food product represent a useful way of understanding and identifying the language and techniques people use. This information can be used for communicating how to prepare a food product through a recipe, to describe the final product, and to provide graphical and step-by-step information. However, the format in which the recipe is presented might be a more important factor evaluated by consumers when deciding on whether to use a recipe or not. Users preferred recipes that had images since they considered them easier and more helpful. On the other hand, they did not like long, very detailed recipes, since they were considered difficult and not helpful. 

## Figures and Tables

**Table 1:** Demographic information of 43 wheat dough preparers and 50 corn dough preparers who participated in the studies.

Demographics	Wheat Study (%)	Corn Study (%)
**Gender**		
Female	79	100
Male	21	0
**Age (years)**		
18–24	5	6
25–44	42	46
45–64	53	44
65 or older	0	4

**Table 2:** Amount of ingredients given to dough preparers.

Ingredient	Wheat (g)	Corn (g)
Flour	500	400
Water	1000	500
Salt	70	50
Instant yeast	14 (approx., equivalent to 2 packets)	NA
Vegetable oil	112	NA

**Table 3:** Demographic information of participants of the online surveys (per study, *n* = 300).

Demographics	Wheat Study (%)	Corn Study (%)
**Gender**		
Female	81	79
Male	19	21
**Age (years)**		
18–24	7	8
25–34	12	14
35–44	17	13
45–54	19	19
55–64	21	24
65 or older	24	22
**Ethnicity**		
White, not of Hispanic origin	84	84
Black, not of Hispanic origin	7	8
Asian or Pacific Islander	4	2
American Indian or Alaskan	1	1
Native Hispanic	3	2
Prefer not to answer	2	3

**Table 4:** Measurement techniques mostly used by 43 wheat dough preparers and 50 corn dough preparers during the dough preparation sessions.

	Wheat (%)	Corn (%)
**Measurement technique**		
Household/Volume	86	18
Weight	5	2
Household/Volume and Weight	9	2
**Not measured ingredients**	84	88
Did not measure flour during mixing	7	80
Did not measure flour during kneading	67	NA
Did not measure the water	9	88
Did not measure oil as an ingredient	40	NA
Did not measure oil to the bowl	12	NA
Did not measure the salt	NA	82

**Table 5:** Amount of each of the ingredients (g) used by 43 wheat dough preparers and 50 corn dough preparers during the dough preparation sessions.

Ingredient	Wheat Dough			Corn Dough		
	Average ^†^ (g)	Minimum (g)	Maximum (g)	Average ^†^ (g)	Minimum (g)	Maximum (g)
Flour	326	193	426	158	43	500
Water	251	191	378	229	52	649
Salt	7	2	36	6	0	52
Yeast	8	4.5	14	NA	NA	NA
Oil	25	0	112	NA	NA	NA

^†^ Amounts used for the recipes.

**Table 6:** Utensils used by 43 wheat dough preparers and 50 corn dough preparers in each of the mixing stages during the dough preparation sessions.

Utensil	Wheat (%)		Corn (%)	
	Dry Ingredients	All (Wet and Dry) Ingredients	Dry Ingredients	All (Wet and Dry) Ingredients
Wooden spoon	44	65	0	0
Metal spoon	19	7	14	16
Rubber spatula	7	9	0	0
Whisk	2	0	0	0
Fork	0	0	0	2
Hands	5	7	16	96
More than one utensil	0	11	0	16

**Table 7:** Mixing and kneading techniques used by 43 wheat dough preparers and 50 corn dough preparers during the dough preparation sessions.

Technique	Wheat (Frequency)			Corn (Frequency)	
	Dry Ingredients	All (Wet and Dry) Ingredients	Kneading	Dry Ingredients	All (Wet and Dry) Ingredients
Stirring/Circular motions	25	31	NA	12	31
From the edges to the center	9	4	NA	1	5
Incorporating all together	8	27	NA	NA	36
Scraping the bowl	2	25	NA	4	15
Folding the mix or the dough	5	12	36	2	19
Well in the center	13	NA	NA	1	NA
Adding water, a little bit at a time	NA	31	NA	NA	17
Breaking lumps	NA	7	NA	NA	8
Adding flour when sticky/wet	NA	12	23	NA	6
Pressing/Pushing/Squeezing the dough	NA	23	43	NA	50
Timewise	NA	2	5	NA	NA
Rolling the dough	NA	NA	12	NA	NA
Quarter turn	NA	NA	25	NA	NA
Using the heel/palms of the hand	NA	NA	19	NA	NA
Flouring the surface	NA	NA	38	NA	NA
Too dry, needs more water	NA	16	NA	NA	20

**Table 8:** Attributes and terms used by 43 wheat dough preparers and 50 corn dough preparers to describe a ready dough.

Attribute	Wheat (Frequency)		Corn (Frequency)
	Dough after Mixing All the Ingredients	Ready Dough (after Kneading)	Ready Dough
Sticky	27	6	NA
One ball/Does not fall apart	25	NA	18
Moist/Wet	11	3	10
Mixed in	21	NA	12
Pulls away from the sides of the bowl	11	NA	NA
Homogeneous/lump free	10	9	20
Hard to stir	9	NA	NA
Soft/Soft but not too soft	7	19	17
Not wet/Dry	7	8	22
Not-sticky	4	30	39
Elastic	NA	32	NA
Smooth	NA	29	3
Consistent	NA	18	NA
Pliable	NA	16	16
One-ball	NA	14	7
Not too hard/Firm	NA	10	17
Airy	NA	10	NA
Can handle with hand/workable	NA	7	20
Spongy	NA	4	NA
Rolls	NA	3	NA
Desired saltiness level	NA	NA	35

## Appendix A

### Appendix A.1. Wheat Recipes

#### Appendix A.1.1. Not Detailed, Step by Step Format

***Makes 2 loaves of bread***

**Initial setup**

- Wash your hands with warm water and soap for at least 20 s.

**Ingredients**

- 3 cups of all purpose wheat flour
- 2 cups of warm water
- 1.5 teaspoon of salt
- 1 packet of instant dry yeast
- 2 tablespoons of vegetable oil

**Dough making procedure**

1. Mix the dry ingredients: yeast, salt and 2/3 of the flour in a bowl.
2. Then, add the oil (if required, as needed), and half of the water.
3. Use a wooden spoon to stir the mix, add the other half of water as needed.
4. Continue stirring with the wooden spoon. When it is ready, turn the dough onto a floured board to knead.
5. Add the rest of the flour as needed.

**Forming and baking procedure**

1. Put the ball of dough in a clean bowl and cover it with plastic wrap.
2. Let the dough rise for 30 min to an hour, until it doubles in size.
3. Grease a loaf pan.
4. Shape the dough into a rectangle about the size of the loaf pan.
5. Put the dough in the pan.
6. Preheat the oven to 425 °F (or 215 °C)
7. Let the dough rest another 30 min to an hour, before baking it.
8. Bake the dough 25–30 min until it is golden brown.
9. Let it cool down for at least 30 min.
10. Remove from the pan.

#### Appendix A.1.2. Very Detailed, Step by Step Format

***Makes 2 loaves of bread***

**Initial setup**

- Wash your hands with warm water and soap for at least 20 s.

**Utensils**

- 1 big bowl
- 1 wooden spoon
- Dry ingredients measuring cups
- Wet ingredients measuring cups
- Thermometer
- Loaf pan

**Ingredients**

Measure the following ingredients one by one.

- 3 cups of all purpose wheat flour split into two
  - 1.5 cups of flour for the initial mixing
  - 1.5 cups of flour to add as needed during mixing and kneading
- 2 cups of warm water (110 °F/43 °C)
- 1.5 teaspoon of salt
- 1 packet of instant dry yeast (7 g)
- 2 tablespoons of vegetable oil

**Dough making procedure (~30 s)**

Mixing dry ingredients

1. In a large bowl add the following ingredients:
  - 1.5 cups of flour
  - 1.5 tsp of salt
  - 1 packet of instant dry yeast
2. Use a wooden spoon:
  - Mix using circular motions, scraping the mix from the sides towards the center.
  - Mix until all the dry ingredients are evenly distributed, use the yeast as a guide since it is a different color.

Mixing of wet and dry ingredients (~4 min)

1. Make a well in the center of the dry ingredients.
2. Add 3/4 cup of the water into the well.
3. Add the oil into the well.
  - Stir the wet and dry ingredients together using the wooden spoon
  - As the dough begins to form, fold the dough into itself as you scrape the mix from the sides to the center incorporating all the ingredients together.
  - Press the dough down to break any lumps.
4. Look at your dough after mixing it:
  - If it looks dry and there is loose flour, add water, 1 tablespoon at a time and continue mixing.
  - If it looks too sticky and there is too much water that is not being incorporated, add 1/4 of a cup of flour and continue mixing.
5. Your dough will be ready when the mixing gets harder and you have one lump-free ball that is sticky, but you can still handle with your hands. The dough should pull away from the sides of the bowl with no liquid left in the bowl and no loose flour.

Kneading (~5–10 min)

1. On your kneading surface (either a cutting board or a counter top), spread ¼ cup of flour, creating a thin layer. Use this flour to cover your hands as well.
2. Scrape the dough out of the bowl onto the floured surface.
3. Start kneading it:
  - Using your hands, roll the dough back and forth over the floured surface, covering it all with flour.
  - With the heels of your hands, push the dough down spreading it away from you, then fold the dough in half and turn it in a quarter turn.
  - Repeat ***step b*** 3 times.
  - If the dough is still sticky, roll it again on the flour that is left on your kneading surface; if you already used all the flour, add another tablespoon of it on the surface, repeat ***steps a and c***.
  - Continue ***steps a to d*** until the dough is no longer sticky.
  - Once the dough is not sticky, repeat ***step b*** until you get a smooth and elastic ball of dough so that when you touch it with one of your fingers, it springs back.
4. Form the dough into a ball.

**Forming and baking procedure**

1. Put the ball of dough in a clean bowl and cover it with plastic wrap.
2. Let the dough rise for 30 min to an hour, until it doubles in size.
3. Grease a loaf pan.
4. Shape the dough into a rectangle about the size of the loaf pan.
5. Put the dough in the pan.
6. Preheat the oven to 425°F (or 215 °C).
7. Let the dough rest another 30 min to an hour, before baking it.
8. Bake the dough 25–30 min until it is golden brown.
9. Let it cool down for at least 30 min.
10. Remove from the pan.

#### Appendix A.1.3. Detailed, Step by Step

***Makes 2 loaves of bread***

**Initial setup**

- Wash your hands with warm water and soap for at least 20 s.

**Utensils**

- 1 big bowl
- 1 wooden spoon
- Dry ingredients measuring cups
- Wet ingredients measuring cups
- Thermometer
- Loaf pan

**Ingredients**

Measure the following ingredients one by one.

- 3 cups of all purpose wheat flour
- 2 cups of warm water (110°F/43°C)
- 1.5 teaspoon of salt
- 1 packet of instant dry yeast (7 g)
- 2 tablespoons of vegetable oil

**Dough making procedure**

Mixing dry ingredients

1. In a large bowl add 1.5 cups of flour, 1.5 tsp of salt and 1 packet of instant dry yeast.
2. Use a wooden spoon to mix the ingredients, until all they are evenly distributed.

Mixing of wet and dry the ingredients

1. Add 3/4 cup of the water and the oil, stir using the wooden spoon.
  - If the dough looks dry and there is loose flour, mix in 1 tablespoon of water.
  - If there is water not incorporated, mix in ¼ cup of flour.
2. Your dough will be ready when you have one lump-free ball that is sticky, but you can still handle with your hands.

Kneading

1. Spread flour on your kneading surface, apply it on your hands and on the surface of the dough.
2. Scrape the dough out of the bowl onto the floured surface.
3. With the heels of your hands, push the dough down, then fold it in half and turn it in a quarter turn; repeat 3 times.
4. If the dough is still sticky, add another tablespoon and continue kneading until it is not sticky.
5. Once the dough is not sticky, continue kneading until you get a smooth and elastic ball of dough.

**Forming and baking procedure**

1. Put the ball of dough in a clean bowl and cover it with plastic wrap.
2. Let the dough rise for 30 min to an hour, until it doubles in size.
3. Grease a loaf pan.
4. Shape the dough into a rectangle about the size of the loaf pan.
5. Put the dough in the pan.
6. Preheat the oven to 425 °F (or 215 °C)
7. Let the dough rest another 30 min to an hour, before baking it.
8. Bake the dough 25–30 min until it is golden brown.
9. Let it cool down for at least 30 min.
10. Remove from the pan.

#### Appendix A.1.4. Detailed, Paragraph Format

***Makes 2 loaves of bread***

**Initial setup**

- Wash your hands with warm water and soap for at least 20 s.

**Dough making procedure**

Mixing dry ingredients: In a large bowl add 1.5 cups of flour, 1.5 tsp of salt and 1 packet of instant dry yeast. Mix them with a wooden spoon, scraping the mix from the sides of the bowl. Mix until all the dry ingredients are evenly distributed.

Mixing of wet and dry the ingredients: Add 3/4 cup of warm water (110 °F/43 °C) and 2 tablespoons of oil, stir using the wooden spoon. If the dough looks dry and there is loose flour, add 1 tablespoon of water; but, if there is water not incorporated, add 1/4 of a cup of flour and continue mixing. Your dough will be ready when you have one lump-free ball that is sticky, but you can still handle with your hands.

Kneading: Use 1/4 cup of flour to spread on your kneading surface, apply on your hands and on the outside of the dough. Scrape the dough out of the bowl onto the floured surface. Using the heels of your hands, push the dough down, then fold the dough in half and turn it in a quarter turn; repeat 3 times. If after this, the dough is still sticky, add another tablespoon of flour on the surface, and continue kneading until it is not sticky. Once the dough is not sticky, continue kneading until you get a smooth and elastic ball of dough.

**Forming and baking procedure**

1. Put the ball of dough in a clean bowl and cover it with plastic wrap.
2. Let the dough rise for 30 min to an hour, until it doubles in size.
3. Grease a loaf pan.
4. Shape the dough into a rectangle about the size of the loaf pan.
5. Put the dough in the pan.
6. Preheat the oven to 425 °F (or 215 °C).
7. Let the dough rest another 30 min to an hour, before baking it.
8. Bake the dough 25–30 min until it is golden brown.
9. Let it cool down for at least 30 min.
10. Remove from the pan.

#### Appendix A.1.5. Detailed, Image Format

***Makes 2 loaves of bread***

**Initial setup**

- Wash your hands with warm water and soap for at least 20 s.

**Utensils**

- 1 big bowl
- 1 wooden spoon
- Dry ingredients measuring cups
- Wet ingredients measuring cups
- Thermometer
- Loaf pan

**Ingredients**

- 3 cups of all purpose wheat flour
- 2 cups of warm water (110 °F/43 °C)
- 1.5 teaspoon of salt
- 1 packet of instant dry yeast (7 g)
- 2 tablespoons of vegetable oil

**Dough making procedure:**

Mixing

4–5 min

Kneading

5–10 min

**Forming and baking procedure**

1. Put the ball of dough in a clean bowl and cover it with plastic wrap.
2. Let the dough rise for 30 min to an hour, until it doubles in size.
3. Grease a loaf pan.
4. Shape the dough into a rectangle about the size of the loaf pan.
5. Put the dough in the pan.
6. Preheat the oven to 425 °F (or 215 °C).
7. Let the dough rest another 30 min to an hour, before baking it.
8. Bake the dough 25–30 min until it is golden brown.
9. Let it cool down for at least 30 min.
10. Remove from the pan.

### Appendix A.2. Corn Recipes

#### Appendix A.2.1. Not Detailed, Step by Step Format

***Makes 3 tortillas***

**Initial setup**

- Wash your hands with warm water and soap for at least 20 s.

**Ingredients**

- 1.5 cups of corn flour
- 1 cup of warm water (110 °F/43 °C)
- 1 teaspoon of salt

**Dough making procedure**

1. Mix the 1 cup of flour and 0.5 teaspoons of salt.
2. Add water a little bit at a time until your dough ready.
3. Add the rest of the flour, salt and water as needed.

**Shaping and cooking procedure**

1. Preheat the skillet (medium heat).
2. Divide the dough into 3 equal-size balls.
3. Cut a plastic bag along the sides and put it in the tortilla press.
4. Put a ball of dough in the middle of the plastic bag in the press.
5. Flattened the tortilla using the tortilla press.
6. Peel the tortilla away from the plastic.
7. Place the tortilla in the skillet and let it cook for 1 min.
8. Turn the tortilla over and let it cook on the other side for another minute.
9. Wrap the tortilla in a warm towel to keep it warm.

#### Appendix A.2.2. Very Detailed, Step by Step Format

***Makes 3 tortillas***

**Initial setup**

- Wash your hands with warm water and soap for at least 20 s.

**Utensils**

- 1 big bowl
- Dry ingredients measuring cups
- Wet ingredients measuring cups
- Measuring spoons
- Thermometer
- A skillet
- A tortilla press
- A zip plastic bag

**Ingredients**

Measure the following ingredients one by one.

- 1.5 cups of corn flour split into two
  - 1 cup of flour for the initial mixing.
  - 1/2 cups of flour to add if needed.
- 1 cup of warm water (110 °F/43 °C)
- 1 teaspoon of salt (you will add 1/2 tsp at a time)

**Dough making procedure** (~3–5 min)

1. In a large bowl add the following ingredients:
  - 1 cup of flour
  - ½ tsp of salt
  - ½ cups of water
2. Use your hands to mix all the ingredients together:
  - Start with a circular movement as you dissolve the flour in the water.
    - If the dough is not forming into a ball and It looks lumpy, dry, and there is loose flour, add a tablespoon of water and continue with ***step 2.***
3. Once everything is coming together into one ball, do one circular motion around the bowl picking up all the flour or dough stuck to the sides of the bowl, then mash the dough against the bowl using your hand.
4. Repeat ***step 3*** until the dough forms one ball that does not stick to the bowl.
5. Take a piece of the dough and make one small ball about the size of an egg.
  - Flatten the ball between your hands, if:
    - The dough sticks to your hands, add another teaspoon of flour and repeat ***steps 2 to 4***.
    - The dough breaks down into pieces or cracks in the middle, add another teaspoon of water and repeat ***steps 2 to 4*** (it is ok if there are small cracks on the edges).
6. Try the taste of the dough, is this the amount of salt you like in your tortillas? If not, add another ½ teaspoon of salt and mix it again as in steps ***2 and 3.***
7. Your dough is ready when it does not stick to the bowl or your hands and is soft, lump-free, and workable.

**Forming and cooking procedure**

1. Preheat the skillet (medium heat).
2. Divide the dough into 3 equal-size balls.
3. Cut a plastic bag along the sides and put it in the tortilla press.
4. Put a ball of dough in the middle of the plastic bag in the press.
5. Flattened the tortilla using the tortilla press.
6. Peel the tortilla away from the plastic.
7. Place the tortilla in the skillet and let it cook for 1 min.
8. Turn the tortilla over and let it cook on the other side for another minute.
9. Wrap the tortilla in a warm towel to keep it warm.

#### Appendix A.2.3. Detailed, Step by Step

***Makes 3 tortillas***

**Initial setup**

- Wash your hands with warm water and soap for at least 20 s.

**Utensils**

- 1 big bowl
- Dry ingredients measuring cups
- Wet ingredients measuring cups
- Measuring spoons
- Thermometer
- A skillet
- A tortilla press
- A zip plastic bag

**Ingredients**

Measure the following ingredients one by one.

- 1.5 cups of corn flour
- 1 cup of warm water (110 °F/43 °C)
- 1 teaspoon of salt

**Dough making procedure**

1. In a large bowl add: 1 cup of flour, 1/2 tsp of salt and 1/2 cup of water.
2. Mix all the ingredients together using your hands.
3. If the dough is not forming into a ball and it looks lumpy and dry, add a tablespoon of water and continue mixing.
4. Once everything is coming together into one ball, pick up all the flour or dough stuck to the sides of the bowl and mash the dough against the bowl using your hand. Continue doing this until the dough is no longer sticky.
5. Take a piece of the dough and flatten it between your hands, if the dough sticks to your hands, add another teaspoon of flour and continue mixing; but, if the dough breaks down into pieces, add another teaspoon of water and continue mixing.
6. Try the dough, if it is needed, add more salt and mix.
7. Your dough is ready when it does not stick to the bowl or your hands, it is lump-free and workable.

**Forming and cooking procedure**

1. Preheat the skillet (medium heat).
2. Divide the dough into 3 equal-size balls.
3. Cut a plastic bag along the sides and put it in the tortilla press.
4. Put a ball of dough in the middle of the plastic bag in the press.
5. Flattened the tortilla using the tortilla press.
6. Peel the tortilla away from the plastic.
7. Place the tortilla in the skillet and let it cook for 1 min.
8. Turn the tortilla over and let it cook on the other side for another minute.
9. Wrap the tortilla in a warm towel to keep it warm.

#### Appendix A.2.4. Detailed, Paragraph Format

**Initial setup**

- Wash your hands with warm water and soap for at least 20 s.

**Dough making procedure**

In a large bowl add 1 cup of flour, 1/2 tsp of salt, and 1/2 cup of water (110 °F/43 °C). Then, mix all the ingredients together using your hands and form a ball. If the ball is not forming and the mix looks lumpy and dry, add a tablespoon of water and continue mixing. Once everything is coming together into one ball, pick up all the flour or dough stuck to the sides of the bowl and mash the dough against the bowl using your hand. Continue doing this until the dough is no longer sticky. Next, take a piece of the dough and flatten it between your hands; if the dough sticks to your hands, add another teaspoon of flour and mix. Likewise, if the dough breaks down into pieces, add another teaspoon of water and mix. Finally, taste the dough. If it is needed, add more salt and mix. Your dough is ready when it does not stick to the bowl or your hands, it is lump-free and workable.

**Forming and cooking procedure**

1. Preheat the skillet (medium heat).
2. Divide the dough into 3 equal-size balls.
3. Cut a plastic bag along the sides and put it in the tortilla press.
4. Put a ball of dough in the middle of the plastic bag in the press.
5. Flattened the tortilla using the tortilla press.
6. Peel the tortilla away from the plastic.
7. Place the tortilla in the skillet and let it cook for 1 min.
8. Turn the tortilla over and let it cook on the other side for another minute.
9. Wrap the tortilla in a warm towel to keep it warm.

#### Appendix A.2.5. Detailed, Image Format

***Makes 3 tortillas***

**Initial setup**

- Wash your hands with warm water and soap for at least 20 s.

**Utensils**

- 1 big bowl
- Dry ingredients measuring cups
- Wet ingredients measuring cups
- Measuring spoons
- Thermometer
- A skillet
- A tortilla press
- A zip plastic bag

**Ingredients**

Measure the following ingredients one by one.

- 1.5 cups of corn flour
- 1 cups of warm water (110 °F/43 °C)
- 1 teaspoon of salt

**Dough making procedure:**

3–5 min

**Forming and cooking procedure**

1. Preheat the skillet (medium heat).
2. Divide the dough into 3 equal-size balls.
3. Cut a plastic bag along the sides and put it in the tortilla press.
4. Put a ball of dough in the middle of the plastic bag in the press.
5. Flattened the tortilla using the tortilla press.
6. Peel the tortilla away from the plastic.
7. Place the tortilla in the skillet and let it cook for 1 min.
8. Turn the tortilla over and let it cook on the other side for another minute.
9. Wrap the tortilla in a warm towel to keep it war.

## Appendix B

**Table A1:** Pearson’s correlation matrix for wheat recipes’ validation (*n* = 300).

Variables	Likeability of Instructions	Format Helpfulness	Ease	Amount of Information	Likelihood to Use It
Likeability of instructions	**1**	**0.805**	**0.632**	**−0.228**	**0.688**
Format helpfulness	**0.805**	**1**	**0.578**	**−0.132**	**0.607**
Ease	**0.632**	**0.578**	**1**	**−0.269**	**0.644**
Amount of information	**−0.228**	**−0.132**	**−0.269**	**1**	**−0.214**
Likelihood to use	**0.688**	**0.607**	**0.644**	**−0.214**	**1**

Values in bold are different from 0 with a significance level alpha = 0.05.

**Table A2:** Pearson’s correlation matrix for corn recipes’ validation (*n* = 300).

Variables	Likeability of Instructions	Format Helpfulness	Ease	Amount of Information	Likelihood to Use It
Likeability of instructions	**1**	**0.768**	**0.656**	−0.100	**0.728**
Format helpfulness	**0.768**	**1**	**0.539**	−0.040	**0.595**
Ease	**0.656**	**0.539**	**1**	−0.211	**0.664**
Amount of information	−0.100	−0.040	−0.211	**1**	−0.093
Likelihood to use it	**0.728**	**0.595**	**0.664**	−0.093	**1**

Values in bold are different from 0 with a significance level alpha = 0.05.

## Appendix C

**Table A3:** ANOVA per cluster, wheat study.

Recipe	Cluster 1 (*n* = 169)	Cluster 2 (*n* = 131)
Images	6.136A	8.076A
Not detailed	5.444 B	7.145B
Detailed	5.408B	7.344BC
Paragraph	4.994C	6.985C
Very detailed	4.385D	6.908C

ABCD: scores with a same letter within a column are not significantly different from each other.

**Table A4:** ANOVA per cluster, corn study.

Recipe	Cluster 1 (*n* = 161)	Cluster 2 (*n* = 139)
Images	5.957a	7.978a
Not detailed	5.329b	7.043b
Detailed	5.155b	7.158b
Very detailed	4.472c	7.101b
Paragraph	4.453c	6.612c

abc: scores with a same letter within a column are not significantly different from each other.
